# Clinical outcomes of endoscopic synovectomy with adjuvant radiotherapy of pigmented villonodular synovitis of the hip: a case series of single center

**DOI:** 10.1186/s12891-022-05141-y

**Published:** 2022-03-02

**Authors:** Hao sun, Xiao-Dong Ju, Hong-Jie Huang, Xin Zhang, Jian-Quan Wang

**Affiliations:** Department of Sports Medicine, Peking University Third Hospital; Institute of Sports Medicine of Peking University; Beijing Key Laboratory of Sports Injuries, 49 North Garden Rd, Haidian District, 100191 Beijing, PR China

**Keywords:** Pigmented villonodular synovitis, Hip endoscopy, Radiotherapy

## Abstract

**Background:**

Though radiotherapy has been widely used for knee pigmented villonodular synovitis (PVNS), there is few literatures about radiotherapy for the treatment of PVNS hip. Thus, the purpose of this study was to analyze the clinical outcomes of endoscopic synovectomy with/without radiotherapy postoperatively of PVNS hip.

**Methods:**

We performed a retrospective study of patients who underwent endoscopy in our hospital from November 2010 to January 2021. Inclusion criteria was patients with magnetic resonance image (MRI) signs, endoscopic findings and/or histological evidence of PVNS. Exclusion criteria was patients lost follow-up. All patients underwent synovectomy endoscopically and were divided into two groups depending on receiving postoperative radiotherapy or not. The primary outcome measurements were the recurrence of PVNS, receiving revision, and/or converting to total hip arthroplasty (THA). The secondary outcome measurements were the patient-reported outcome (PRO) collected at pre- and post-operation, which consist of Hip Outcome Score Activities of Daily Living (HOS-ADL), modified Harris Hip Score (mHHS), International Hip Outcome Tool-12 (IHOT-12), Non-arthritic Hip Scale (NAHS), and visual analog scale (VAS).

**Results:**

In a case series of 16 patients (8 cases of male, 50%), 4 (25%) cases were localized type and 12 (75%) cases were diffuse type. The average follow-up was 44.8 ± 38.2 months (range,3 to 110). 8 (50%) cases (6 diffuse cases and 2 localized cases) received radiotherapy postoperatively, and the rest (6 diffuse cases and 2 localized cases) received endoscopic treatment alone. At the latest follow-up, 3 (18.75%) cases (2 diffuse cases and 1 localized case) who did not receive radiotherapy converted to arthroplasty. The preoperative HOS-ADL, mHHS, IHOT-12, NAHS, VAS scores of remaining 13 patients were 63.1 ± 19.1 (range,32.0 to 98.8), 54.8 ± 20.1 (range, 10.0 to 77.0), 50.9 ± 15.4 (range, 31.0 to 76.6),51.6 ± 15.9 (range, 20.0 to 84.4), 6.0 ± 1.4 (range,4.0 to 8.0) points, respectively. The latest HOS-ADL, mHHS, IHOT-12, NAHS, VAS scores of the 13 patients were 79.7 ± 10.8 (range, 58.0 to 97.6), 78.6 ± 9.1 (range,55.0 to 87.0), 74.7 ± 9.7 (range, 55.6 to 91.0), 78.9 ± 18.7 (range,20.0 to 92.5), 3.1 ± 1.2 (range,2.0 to 6.0) points respectively.

**Conclusion:**

Endoscopic synovectomy can achieve satisfactory PRO in PVNS hip patients. Besides, postoperative adjuvant radiotherapy can achieve higher hip survivability than synovectomy alone in this present study.

## Background

Pigmented villonodular synovitis (PVNS) is a rare, benign disease that is characterized by destructive and recurrent. This disease was first documented by Chassaignac [1]. in 1852, and the lesion was found to be in the flexor tendon sheath of the finger. Later, Jaffe et al. [2] established the pathology of PVNS in 1941. PVNS can not only affect the joints but also can invade peripheral structures including muscle, tendon, bone and skin [3, 4]. The affected joint is mainly distributed in the knee (80%) followed by the hip (15%) [5, 6]. The incidence of PVNS is estimated at 1.8 per million persons [7]. Though PVNS is mostly occurs in middle-aged patients [7], a retrospective multicenter study [8] reported that the youngest case was of a two-year-old, and the oldest, 83 years.

PVNS has no specific symptoms, usually manifesting hemorrhagic effusion, swelling, pain, and consequent movement restriction, which might be misdiagnosed. Nowadays, the development of magnetic resonance imaging (MRI) has become an effective and instructive method for diagnosing PVNS. PVNS can be divided into two types [7, 9]: diffuse type and localized type. The localized PVNS is characterized by focal involvement of the synovium, with either nodular or pedunculated masses; the diffuse form involves almost the entire synovium. Both types can occur intra- and/or extra-articular. Although the biology of those two forms is similar, diffuse form is more aggressive clinically [9]. For the treatment of PVNS, both opening and endoscopic management can gain satisfactory patient-reported outcomes. Due to the tumor-like character [10, 11] and high recurrence [8] of PVNS, radiotherapy was used widely after synovectomy.

To our best knowledge, this is the first case series study that aim to analyze the clinical outcomes of endoscopic synovectomy with/without radiotherapy postoperatively of PVNS hip.

## Method

**Fig. 1:**
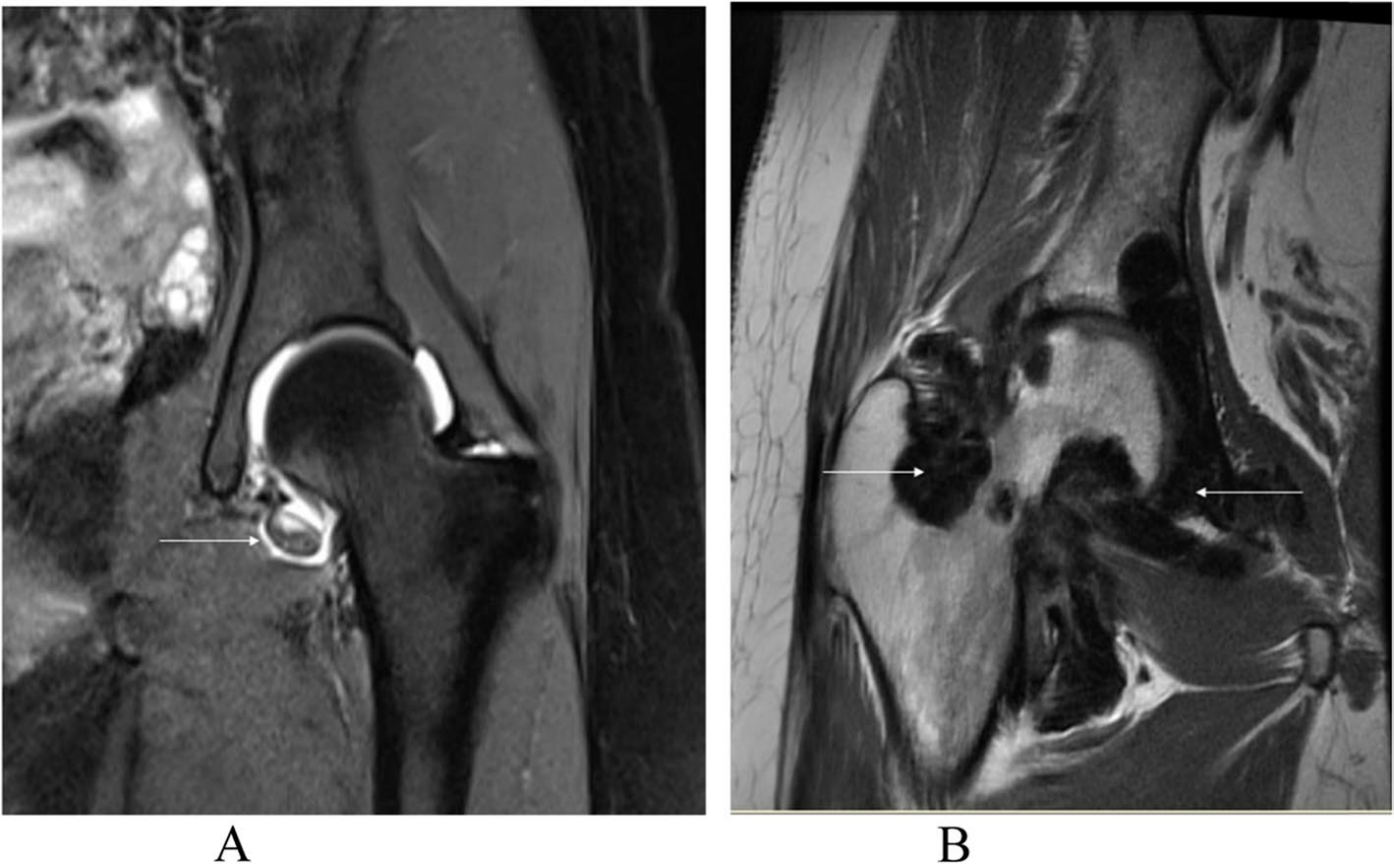
MRI findings of diffuse or localized type of PVNS. **A**: MRI coronary PD-weighted image of the left hip of localized PVNS, a wide band of short T2 signal nodule can be seen medial to the articular space. **B**: MRI coronary T1-weighted image of the right hip of diffuse PVNS, multiple long T1 mixed T2 signal shadows can be seen in the articular space. White arrows indicate the lesion sites

**Fig. 2:**
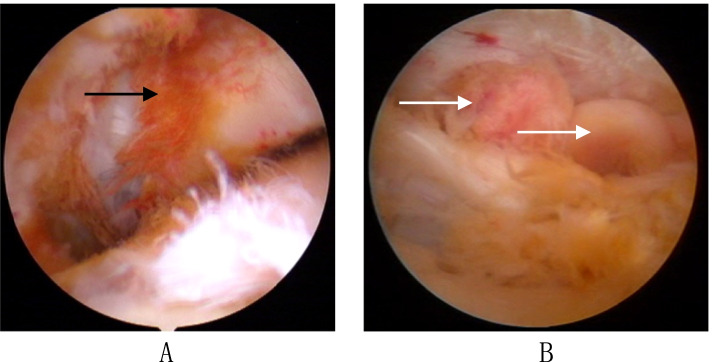
Intraoperative finding of diffuse or localized type of PVNS. Diffuse vs localized (nodular) PVNS lesions. Arthroscopic view of PVNS lesions, which can present as (**A**) diffuse PVNS lesions (black arrow) to (**B**) localized (nodular) PVNS lesions (white arrow)

**Fig. 3:**
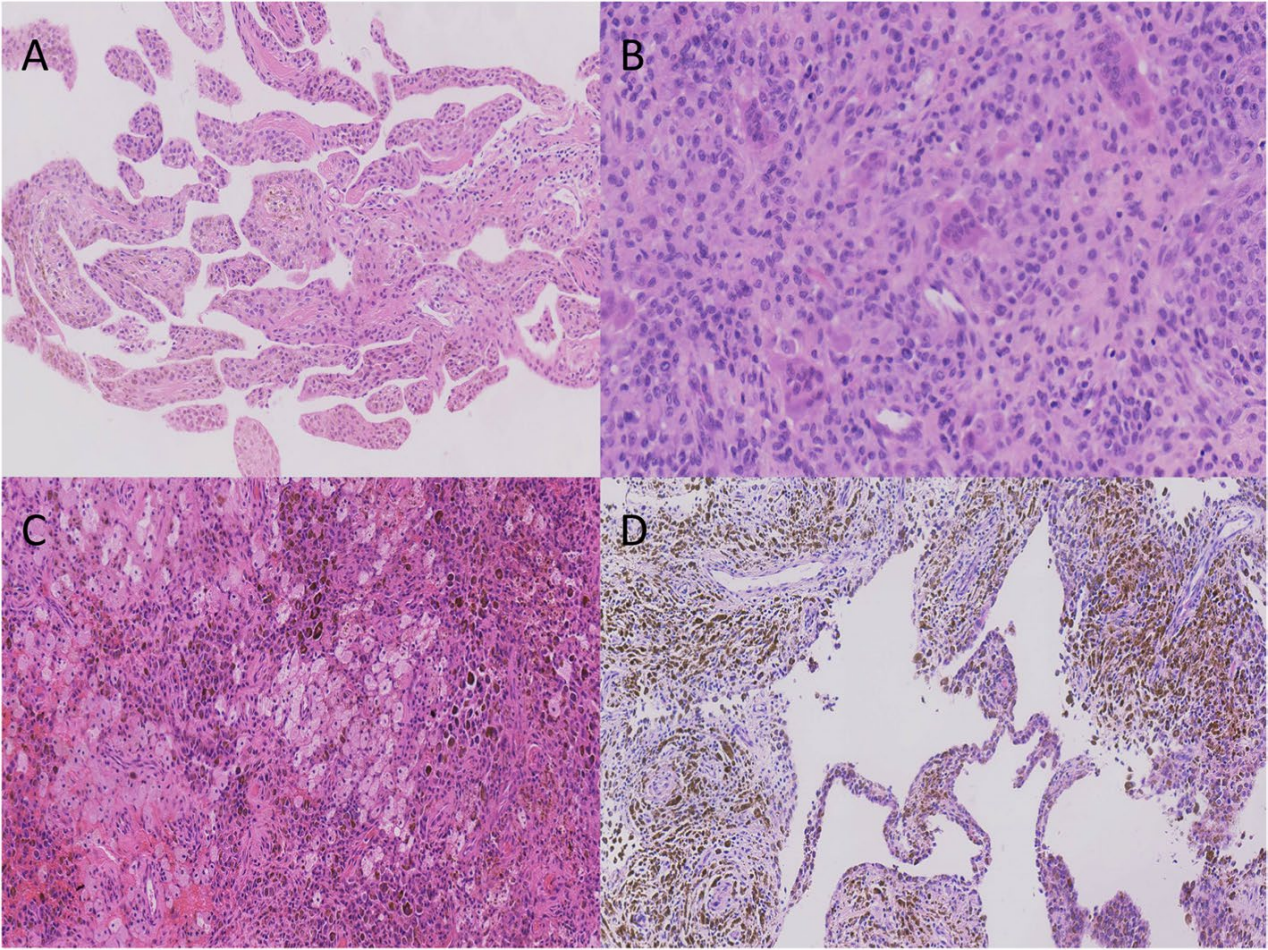
different histopathological features of PVNS. **A**. Large amount of villous synovial hyperplasia (HE× 20). **B**. Homogeneous monocytes associated with scattered multinucleated giant cells (HE× 40). **C**. Lots of hemosiderin and foam cells (HE× 20). **D**. Synovial villous hyperplasia and abundant hemosiderin deposition (HE× 20)

This study was a retrospective case series in which all patients underwent arthroscopy at our hospital from November 2010 to February 2021.Inclusion criteria was patients with MRI signs, endoscopic findings, and/or histological evidence of PVNS (Figs. 1 and 2 shows the type of PVNS, Fig. 3 shows the histopathological features). Exclusion criteria was patients lost follow-up. All patients underwent a standard preoperative imaging examination, including hip radiograph and MRI. Basic demographic information, such as age, sex, BMI, symptom duration time, and follow-up period were collected. The degenerative change was evaluated according to Tönnis grading system [12] on an anteroposterior radiograph. Operative treatment for concomitant pathologies, such as femoroacetabular impingement or labral tear, was also recorded. Cartilage injury of the femoral head and acetabulum were recorded intraoperatively according to the Outerbridge grading system [13]. The size of resected synovium was collected (detailed information in Table 1). Considering the aggressive nature of PVNS, postoperative radiotherapy is recommended. Among patients who did not receive radiotherapy, the main reason was concern about radiotherapy toxicity. For patients who received radiotherapy, the clinical target volumes were defined to cover 5 mm around the current or original lesion sites. Details of radiotherapy included the using dose, fraction, and the time interval after the operation (Table 2). Radiotherapy was administrated in one session of 7 patients and two sessions of 1 patient. The primary outcome measurements were the recurrence of PVNS, receiving revision, and/or converting to total hip arthroplasty. The secondary outcome measurements were the patient-reported outcome collected at pre- and post-operation, which consist of Hip Outcome Score Activities of Daily Living (HOS-ADL), modified Harris Hip Score (mHHS), International Hip Outcome Tool-12 (IHOT-12), Non-arthritic Hip Scale (NAHS), and visual analog scale (VAS) [14].

**Table 1:** Case series patients’ detail

patient	Age,y	Sex	BMI,kg/m^2^	Symptom duration,m	Follow-up time,m	misdiagnosis	anesthesia	Surgical detail	Cartilage injury grading of acetabulum	Cartilage injury grading of Femoral head	type	Specimen size, mm	Radiotherapy	recurrence or Conversion/ time after surgery
1	27	M	22.34	10	124	–	lumbar anesthesia	–	0	III	localized	lost	–	THA,55 m
2	23	F	29.37	16	117	Rheumatoid arthritis	General anesthesia	–	II	III	diffuse	lost	–	THA,87 m
3	16	M	23.51	1	110	inflammation	General anesthesia	–	0	III	diffuse	11*8*2	yes	–
4	21	F	20.08	1	94	–	lumbar anesthesia	–	0	I	localized	30*15*5	yes	–
5	24	M	22.40	18	90	Synovial chondromatosis	General anesthesia	Femoral osteoplasty	0	II	diffuse	25*20*10	yes	–
6	19	M	18.21	1	82	–	General anesthesia	Femoral osteoplasty	0	II	diffuse	10*8*2	yes	–
7	66	F	27.34	8	54	–	General anesthesia	Labral debridement	0	II	localized	30*35	–	–
8	36	M	25.95	60	43	–	General anesthesia	Labral debridement	0	II	diffuse	15*8*2	yes	–
9	29	M	24.34	6	40	–	General anesthesia	–	IV	IV	diffuse	30*30*5	–	–
10	24	F	20.32	24	34	Synovial chondromatosis	General anesthesia	Femoral osteoplasty Acetabuloplasty, Labral repair,	0	I	localized	Diamete*r* = 2	yes	–
11	18	F	20.20	6	27	Osteonecrosis of the Femoral Head	lumbar anesthesia	Labral repair	IV	III	diffuse	Diamete*r* = 8	–	THA,8 m
12	24	F	16.22	36	17	–	General anesthesia	–	IV	IV	diffuse	10*6*3	–	–
13	29	F	22.04	9	8	–	lumbar anesthesia	Femoralosteoplasty,Acetabuloplasty, Labral repair	IV	IV	diffuse	23*15*2	–	–
14-	23	F	17.56	30	5	–	lumbar anesthesia	–	IV	IV	diffuse	15*10*5	–	–
15	40	M	20.76	12	3	–	General anesthesia	–	III	I	diffuse	15*10*3	yes	–
16	33	M	24.96	14	3	–	lumbar anesthesia	–	0	IV	diffuse	10*10*2	yes	–

*y* Year, *m* Month, *mm* Millimeter, *THA* Total hip arthroplasty

*F* Female, *M* Male

**Table 2:** Patients’ radiotherapy detail

patients	Lesion type	Radiotherapy dose/fraction	First treatment interval after surgery, m	complications
3	diffuse	CRT:20Gy/10f	24	none
4	localized	IMRT:12Gy/6f	1	none
5	diffuse	CRT:20Gy/10f	2	none
6	diffuse	CRT:20Gy/10f	0	none
8	diffuse	CRT:20Gy/10f,	1	none
10	localized	CRT:20Gy/10f	2	none
15	diffuse	IMRT:10Gy/5f	1	none
16	diffuse	IMRT:30Gy/15f	1	none

*m* Month, *CRT* Conformal radiotherapy, *IMRT* Intensity modulated radiotherapy

### Statistical analysis

Statistical analysis was performed using SPSS software, version 23.0. Categorical variables are represented as a number and percentage, whereas continuous variables are represented by average, standard deviation, and range.

## Results

We retrospectively collected 16 hip PVNS patients (8 cases of female, 50%) who underwent endoscopy at our hospital. Among those patients, 1 case was misdiagnosed as inflammation, 1 case as rheumatoid arthritis, 1 case as osteonecrosis of the femoral head, 2 cases as synovial chondromatosis, in other medical facilities previously. There were 4 (25%) cases of localized type and 12 (75%) cases of diffuse type. All patients underwent synovectomy endoscopically, among which 8 (6 diffuse cases and 2 localized cases,50%) patients received radiotherapy postoperatively. For the femoral head cartilage injury, 5 cases were degree IV, 4 cases were degree III, 4 cases were degree II, and 3 cases were degree I. And for the acetabular cartilage injury, 5 cases were degree IV, 1 case was degree III, 1 case was degree II, 9 cases had no cartilage injury. All injuries were located at anterosuperior sites of femoral head or acetabulum.

Considering the patients’ condition comprehensively, we would recommend adjuvant radiotherapy after surgery. For these patients who didn’t receive radiotherapy, most of the reason could be attributed to concerns about the potential effect of radiotherapy, such as fracture, stiffness, carcinoma, and impotence. We, therefore, grouped patients into two groups based on whether they received radiotherapy (RT group) or not (NRT group). Due to long interval after surgery (24 months) of a patient, the patient was not included in the RT group for statistical analysis. At the latest follow-up, no recurrence or revision were in those patients, but 3 cases (2 cases of diffuse type and 1 case of localized type) of NRT group converted to THA (Detail in Table 1). All of them had severe multiple bone destruction due to progressing osteoarthritis. For the remaining 13 cases, the mean operative age was 29.5 years (range, 16 to 66). The mean BMI was 21.8 kg/m^2^ (range,16.2 to 27.3). The mean follow-up was 44.8 months (range,3 to 110). The symptom duration time before the operation was 16.9 months (range,1 to 60) (Detail in Table 3).

**Table 3:** Demographic characteristics of patients at the latest follow-up

	Total(*n* = 13)		Radiotherapy(*n* = 7)		Non-radiotherapy(*n* = 5)	
	Pre	Post	Pre	Post	Pre	Post
HOS-ADL	63.1 ± 19.1 (32.0–98.3)	79.7 ± 10.8 (58.0–97.6)	61.1 ± 21.0 (32.0–97.3)	81.4 ± 13.4 (58.0–97.6)	69.3 ± 17.3 (57.4–98.8)	76.2 ± 6.8 (67.7–85.0)
mHHS	54.8 ± 20.1 (10.0–77.0)	78.6 ± 9.1 (55.0–87.0)	52.4 ± 25.4 (10.0–77.0)	81.4 ± 6.3 (72.0–87.0)	62.2 ± 8.1 (52.0–71.0)	75.0 ± 12.5 (55.0–87.0)
iHOT-12	50.9 ± 15.4 (31.0–76.6)	74.7 ± 9.7 (55.6–91.0)	55.9 ± 17.0 (31.0–76.6)	75.3 ± 11.2 (55.6–91.0)	45.2 ± 13.5 (35.0–68.3)	73.1 ± 9.3 (64.8–84.0)
NAHS	51.6 ± 15.9 (20.0–84.4)	78.9 ± 18.7 (20.0–92.5)	55.1 ± 16.3 (35.8–84.4)	83.0 ± 7.1 (72–92.5)	46.9 ± 17.8 (20.0–70.0)	73.0 ± 30.0 (20.0–91.3)
VAS	6.0 ± 1.4 (4.0–8.0)	3.1 ± 1.2 (2.0–6.0)	6.3 ± 1.1 (5.0–8.0)	3.1 ± 0.9 (2.0–4.0)	6.0 ± 1.6 (4.0–8.0)	3.2 ± 1.6 (2.0–6.0)

*y* Year, m Month

The preoperative HOS-ADL, mHHS, IHOT-12, NAHS, VAS scores of remaining 13 patients were 63.1 ± 19.1 (range,32.0 to 98.8), 54.8 ± 20.1 (range, 10.0 to 77.0), 50.9 ± 15.4 (range, 31.0 to 76.6),51.6 ± 15.9 (range, 20.0 to 84.4), 6.0 ± 1.4 (range,4.0 to 8.0) points, respectively. The latest HOS-ADL, mHHS, IHOT-12, NAHS, VAS scores of the 13 patients were 79.7 ± 10.8 (range, 58.0 to 97.6), 78.6 ± 9.1 (range,55.0 to 87.0), 74.7 ± 9.7 (range, 55.6 to 91.0), 78.9 ± 18.7 (range,20.0 to 92.5), 3.1 ± 1.2 (range,2.0 to 6.0) points, respectively. (Detail in Table 4).

**Table 4:** PRO at latest follow-up by radiotherapy or not

	Total(*n* = 13)		Radiotherapy(*n* = 7)		Non-radiotherapy(*n* = 5)	
	Pre	Post	Pre	Post	Pre	Post
HOS-ADL	63.1 ± 19.1 (32.0–98.3)	79.7 ± 10.8 (58.0–97.6)	61.1 ± 21.0 (32.0–97.3)	81.4 ± 13.4 (58.0–97.6)	69.3 ± 17.3 (57.4–98.8)	76.2 ± 6.8 (67.7–85.0)
mHHS	54.8 ± 20.1 (10.0–77.0)	78.6 ± 9.1 (55.0–87.0)	52.4 ± 25.4 (10.0–77.0)	81.4 ± 6.3 (72.0–87.0)	62.2 ± 8.1 (52.0–71.0)	75.0 ± 12.5 (55.0–87.0)
iHOT-12	50.9 ± 15.4 (31.0–76.6)	74.7 ± 9.7 (55.6–91.0)	55.9 ± 17.0 (31.0–76.6)	75.3 ± 11.2 (55.6–91.0)	45.2 ± 13.5 (35.0–68.3)	73.1 ± 9.3 (64.8–84.0)
NAHS	51.6 ± 15.9 (20.0–84.4)	78.9 ± 18.7 (20.0–92.5)	55.1 ± 16.3 (35.8–84.4)	83.0 ± 7.1 (72–92.5)	46.9 ± 17.8 (20.0–70.0)	73.0 ± 30.0 (20.0–91.3)
VAS	6.0 ± 1.4 (4.0–8.0)	3.1 ± 1.2 (2.0–6.0)	6.3 ± 1.1 (5.0–8.0)	3.1 ± 0.9 (2.0–4.0)	6.0 ± 1.6 (4.0–8.0)	3.2 ± 1.6 (2.0–6.0)

*RT* Radiotherapy, *NRT* Non-radiotherapy

## Discussion

Although PVNS occurs most commonly in larger joints, it can still arise in other parts of the body, which can be seen in the spine, temporomandibular joint [15, 16]. Regardless of where PVNS occurs, the traditional treatment is synovectomy, which can be done by opening or endoscopic surgery. Although the opening method has a wider field of view than endoscopic surgery, which can bring the advantage of relatively complete debridement of diseased synovial tissue, it is more invasive than the endoscopic technique. Both two treatments can achieve satisfactory PRO from short- to long-term follow-up [17–19]. A case series study of 14 hips PVNS conducted by Nazal et al. [19] indicated that endoscopic management was an effective method with a survival rate of 93% (13/14), 1 (7%) recurrence, and 0 arthroplasty. A retrospective study of 13 hip PVNS cases that underwent arthroscopy reported only 1 case converted to THA at 6 years postoperatively because of progressive osteoarthritis [17]. However, a retrospective study by Schwartz [20] found that the recurrence of treatment for PVNS endoscopically is higher than open surgery, especially for diffuse type. The higher failure rate of treatment for hip PVNS might be explained by periarticular destruction within the closure capsule and the difficulty of surgical resection [21].

Mankin et al. [21] put forward that total hip arthroplasty was the only treatment choice by analyzing 12 cases of hip PVNS treated from 1972 to 2009. However, a retrospective study conducted by Tibbo et al. [22] of case series of 25 PVNS patients who underwent arthroplasty found that the 5- and 10-year survivorship free from any revision were 83 and 63%, respectively. However, following the THA, 19 patients (76%) sustained at least 1 complication, most commonly aseptic loosening. Besides, another retrospective study of 16 hip PVNS patients underwent arthroplasty with an average follow-up of 16.7 years conducted by Vastel et al. [23] reported 1 case of recurrence and 9 cases of revision. Although this suggests that arthroplasty for hip PVNS had a comforting recurrence rate, the higher rate of complication in arthroplasty might make endoscopic treatment the preferred choice. In our study, due to continuing progression of osteoarthritis, 3 cases converted to THA eventually. But, compared with other patients’ status in our study, patients with more severe joint injury were not converted to THA at the latest follow-up. Previous joint damage might be attributed to secondary injury or PVNS, however, in most conditions, it is hard to explain the outcome when trying to determine whether the symptom might be due to recurrent disease or progression of secondary joint damage [17]. The secondary damage cannot be reversed, nor can it prevent the progression of osteoarthritis. Therefore, based on the condition of affected joints, the surgeon must fully consider the decision to perform less invasive endoscopic surgery or perform total hip arthroplasty.

Because of the infiltrative nature and incomplete resection of PVNS, postoperative adjuvant therapy, such as brachytherapy injection, or external beam radiation, was recommended after resection, especially for diffuse-type PVNS [24]. However, Stephan et al. [25] did not suggest postoperative adjuvant radiotherapy for PVNS patients because of the possible toxic properties of radiotherapy.

A retrospective study of 14 knee PVNS patients who underwent radiotherapy showed 11 patients had good or excellent limb outcomes [26]. A study of 7 PVNS patients (5 knees, 1 hip, 1 wrist) who underwent radical surgery and postoperative radiotherapy showed that 6 patients had asymptomatic limb function and excellent quality of life at average 29 months follow-up [27]. Besides, Horoschak et al. [24] conducted a retrospective study of 17 PVNS patients with 18 lesion sites (12 sites of knee, 3 sites of ankle, 2 sites of hand, and 1 site of spine) treated with postoperative radiotherapy and found that the initial local control rate was 75% with an average follow-up of 46 months. Furthermore, a study demonstrated that 41 of 50 PVNS patients (20 cases of knee, 9 cases of ankle, 7 cases of foot, 6 cases of hand, 4 cases of hip,4 cases of wrist) underwent radiotherapy after surgical resection can gain long-term (mean follow-up period of 94 months) good/excellent functio n[28]. No complication or serious complication was found in any of the above studies. It follows that, for the treatment of PVNS patients, postoperative adjuvant radiotherapy might be an effective management that can achieve satisfactory short- to long-term prognosis with no severe complication. However, the optimal dose used for the treatment of PVNS is unclear now. Some studies reported that radiotherapy for PVNS using low dose as 16–20 Gy can achieve the outcome of no recurrence [9], whereas other studies using dose as high as 50 Gy with no complications [26, 27]. In our study, the using dose for PVNS was 10–30 Gy with no complication.

With the persistent development of radiotherapy, the adjuvant method has developed from traditional treatment to three-dimensional conformal radiotherapy(3D-CRT), intensity modulated radiotherapy (IMRT), and other novel radiotherapy technologies. Those emerging technologies have the advantages of precise positioning and accurate design.

Radiotherapy has been used widely for refractory cases in the knee joint, but there are few cases in the setting of radiotherapy of hip PVNS exist in the contemporary literature. To our best knowledge, this is the first study aimed to compare the clinical outcomes of hip PVNS patients who underwent CRT or IGRT followed by synovectomy endoscopically with those who received isolated synovectomy. In this study, we found a higher rate of hip joint survivability of the RT group than NRT group, which might provide the evidence that adjuvant radiotherapy treatment after endoscopic synovectomy for hip PVNS can be an effective and safe method.

### Limitation

There are several important limitations of this study. First, though the size of the sample is relatively large for known reports of a single center, the total sample size was still small, and there is a bias in patients’ choice of radiotherapy, thus more patients were needed to compare the results. Second, though satisfactory average mid-term PRO gained in our study, the long-term outcome remains to be seen. Third, the time span of this study was more than 11 years, which may affect the outcomes because of the improvement of surgery and radiotherapy technology. Fourth, treatment for concomitant pathology were performed, which made it difficult to distinguish whether the improved PRO was due to the treatment of PVNS or other hip lesions.

## Conclusion

Endoscopic synovectomy can achieve satisfactory PRO. Besides, postoperative adjuvant radiotherapy can achieve higher hip survivability than synovectomy alone in this present study.

## Data Availability

The datasets used and/or analyzed during the current study are available from the corresponding author on reasonable request.

## Abbreviations

PVNS: Pigmented villonodular synovitis
MRI: Magnetic resonance image
THA: Total hip arthroplasty
PRO: Patient-reported outcome
HOS-ADL: Hip Outcome Score Activities of Daily Living
mHHS: Modified Harris Hip Score
IHOT-12: International Hip Outcome Tool-12
NAHS: Non-arthritic Hip Scale
VAS: Visual analog scale
CRT: Conformal radiotherapy
IMRT: Intensity modulated radiotherapy
RT: Radiotherapy
NRT: Non-radiotherapy
